# Identity Multiplicity in an Ethnic and Religious Minority in Latvia: Old Believer Youth

**DOI:** 10.3389/fsoc.2021.641622

**Published:** 2021-03-29

**Authors:** Anita Stasulane

**Affiliations:** Institute of Humanities and Social Sciences, Daugavpils University, Daugavpils, Latvia

**Keywords:** ethnic minority, religious minority, ethnic identity, religious identity, national identity, European identity, Old Believers, youth

## Abstract

The paper explores the relationship between the religious identity and the ethnic and national identities of Old Believer youth in Latvia. This case is of particular interest in providing an in-depth insight into the intersection of ethnicity, nationality and religion, as the Old Believers are an ethnic and religious minority living in Latvia. Applying the concepts of multiple identities, this article explores the role of religion played in the integration of identity among young people belonging to the Old Believer religious community: their self-understanding as a composition of intersecting identities that influence each other; the manifestations of the intersection of various identities; the relationship of identity integration to religion. The analysis is based on the findings of ethnographic research conducted in the Old Believer youth group in Daugavpils (Latvia) within a framework of the international project “Cultural Heritage and Identities of Europe’s Future”, funded from the European Union’s Horizon 2020 Research and Innovation Program under Grant Agreement No. 770464. The author has highlighted that today’s rapid changes are leading to identity crisis: an individual faces difficulty in shaping and maintaining a stable identity, since economic life is becoming increasingly unpredictable and communities are becoming fragmented. The identity of Latvian Old Believer youth forms and develops in a local cultural context, and is affected by the social change. The qualitative data collected during the fieldwork provided a useful resource for an analysis of belonging, the crucial factor in the formation of identity for Old Believer youth. As the voices of young people in this study reveal, three types of belonging characterize Old Believer youth: their ethnicity, which interacts with national belonging in a complex way; the local belonging, which is stronger than the global one; the European belonging, which conflicts with national belonging and ethnicity.

## Introduction

In Latvia, there is a group of the population that have become ethnic minority literally over night when during the fall of the USSR the Republic of Latvia was re-established. This group located in the boarder region between Europe and Russia is particularly relevant for researching identities. The young people of the Russian ethnic minority in Latvia is of special interest since there is a reason to assume that they have multiple identities (Bauman, 2004; Waechter, 2016), i.e., regional, ethnic, national and religious at the same time. Furthermore, those identities are believed to be intertwined (Risse, 2010; Delanty, 2003).

In the Latvian political and economic environment, the dominant view is that the identities of ethnic minorities should be integrated into the national identity, which has the Latvian language and culture at its core. Latvian law guarantees equal rights to all ethnic groups living in Latvia, among them, their rights to cultural autonomy and cultural self-determination. This includes rights to establish their own societies, to celebrate their ethnic community’s festivals, use their own symbols, to maintain contacts with their compatriots in other countries, to develop professional and amateur art etc. (Likumi, 1991). However, in pre-election political discourse, the interests of ethnic minorities are often placed in confrontational positions, setting off Russians against Latvians, in particular.

The research undertaken in the Old Believer community continues analysis of the cultural autonomy and identity of ethnic and religious minorities which commenced in the 1990s, when researchers attempted to find out the degree to which national and civic values were expressed in the consciousness of representatives of ethnic groups and how they interacted with attempts to preserve ethnic cultural values. There is particularly active research taking place on the Russian ethnic minority: comparative case studies of post-Soviet diasporas (Melvin, 1995) and ethnographic research (Laitin, 1998), applying social-psychological approaches (Hagendoorn et al., 2001), structuralist arguments (Galbreath, 2005) and a discourse-theoretical approach (Cheskin, 2015) is being undertaken on a regular basis. The research being carried out by Latvian sociologists, political scientists, ethnographers, and historians (Apine and Volkovs, 1998; Dribins, 2007; Dribins, 2008; Makarovs and Boldāne, 2008; Mierina et al., 2017) is also significant.

Within a framework of the international project “Cultural Heritage and Identities of Europe’s Future” (CHIEF), 8 months of fieldwork was conducted with a Latvian Old Believer youth group in Daugavpils, Latvia’s second largest city. This case is of particular interest, providing an in-depth insight into the intersection of ethnicity, nationality and religion, as the Old Believers are an ethnic and religious minority living in Latvia. The goal of fieldwork was to investigate cultural experiences and attitudes of youth towards local cultural heritage as well as the cultures of other peoples and countries by applying ethnographic research methods. The main attention of this paper is concentrated to the identity multiplicity of Old Believer youth. It focuses on the research questions of how the group of the Old Believers youth is structured (membership, hierarchy, forms of activities), how they describe their ethnicity, nationality and belonging to Europe, how specifically these identities overlap with each other, how the multiplicity of identities impacts self-categorization of themselves as “European”. This study can spark novel questions on the patterns of relationships among multiple identities.

It might be important to note that in this paper, the ethnicity and ethnic identity refer to the common cultural features that a group of people share including a common language, common customs, a belief in a common genealogical descent and ties with a specific territory (Meer, 2014). As Smith points out, a nation is a named and self-defining human community whose member cultivate shared memories, symbols, myths, traditions and values, inhabit and are attached to historic territories or “homelands”, create and disseminate a distinctive public culture, and observe shared customs and standardized laws (Smith, 1991; Smith, 2002).

Data collected by undertaking observations in the group and by interviewing young people of various ages (from 13 to 24 years of age) revealed the role of religion played in the identity integration of young people belonging to the Old Believer religious community: their self-understanding as a composition of intersecting identities that influence each other; the manifestations of the intersection of various identities; the relationship of the integration of identity with religion. This study on the Latvian Old Believer youth enriches the understanding of young people’s identity multiplicity, which are embedded in local contexts and driven by young people’s own perception of belonging.

## Historical Background

A brief description of the Old Believers community’s history is required to understand what has facilitated the formation and presence of this ethnic and religious minority in Latvia. The Old Believers, as a religious group, split off from the Russian Orthodox Church, and arrived in Latvia from Russia. They did not accept the canonic and liturgical reforms implemented in the Russian Orthodox Church in the mid-seventeenth century. Those who wished to remain faithful to “the old rite” and opposed the adaptation of the Russian Orthodox Church practices to the practices of the Greek Orthodox Church, were subject to repression. This caused the defenders of “the old rite” to flee to outlying parts of Russia and also beyond its borders.

Groups of Old Believers reached the western part of today’s Latvia in 1659, which at that time was the Duchy of Courland (Podmazovs, 2001, p. 51). The first wave of immigration by the Old Believers lasted till the end of the seventeenth century and was characterized by the favorable attitude towards the arrivals by institutions of local authority and the nobility. The Duchy of Courland had been laid waste by wars with the Russians, Poles and Swedes. The number of population had been reduced significantly by epidemics of the plague (1657–1661) and the land lay uncultivated in many places, which is why landlords were interested in the influx of a new labor force. The Old Believers settled in remote places in rural areas, where they engaged in agriculture, fishing, crafts and trades.^1^ The difficult living conditions required intensive work, resulting in the development of a social personality type that was very patient, thrifty, prudent, tenacious and skilled in business.

A particularly fortuitous situation developed for the Old Believers in the eastern part of Latvia, in Latgale (a part of Rzeczpospolita at that time), because the king of Polish-Lithuanian Commonwealth had issued an order about “secessionists being free to remain in Polish regions”^2^ (Lileev, 1895, p. 121). The Old Believers settled in Liginišķi^3^ in 1660, where they soon built a church (Podmazovs, 2001, p. 51). The Old Believers also settled in Riga in the early eighteenth century. Conditions for the Old Believers remained unchanged when Russian troops invaded the central part of Latvia, Vidzeme and Riga, because the local administration remained in the hands of the German nobility. The Old Believer communities could continue their lives in Latvia without any real obstacles right up until the nineteenth century, with the families of rich Old Believer traders and entrepreneurs developing. The situation changed after 1825, when a serious crackdown^4^ commenced against Old Believers in the Russian Empire, which also affected the territory of what is today Latvia. The Russian Orthodox Church was used as an instrument in the battle against the Old Believers. Even though this policy affected the lives of the Old Believers in a negative way (only the Orthodox doctrine was taught in schools and many Old Believer churches were closed down), they were able to preserve their community, due to their faith in their religious traditions and their favorable economic position.

The Old Believers regained the freedom to practice their religious traditions after the Revolution of 1905. After the issuing of the tsar’s manifesto declaring religious freedom, the Old Believers were allowed to build and restore their churches and to establish congregations, societies, schools etc. This initiated a revival for Old Believers in Latvia too, but just like the representatives of other ethnic and religious communities, the Old Believers were also drawn into the rapid socio-political changes of the twentieth century, which were brought by World War I, the Russian Revolution of 1917 and the Latvian War of Independence (1918–1920) etc.

After the proclamation of the Republic of Latvia (1918), the Old Believers participated in Latvian political life and implemented cultural and educational initiatives. Up until World War II, the majority of the Russian population of Latvia were Old Believers: census data shows that 107,195 Old Believers lived in Latvia in 1935 (Salnītis, 1936, p. 86). Even though the majority were farmers, small traders or workers, there were also important businessmen and politicians among them, who were elected repeatedly to the Latvian Parliament.

Despite internal disharmony, the Old Believers community was able to pull together and developed successfully until 1940, when the USSR occupied Latvia. The most prosperous and well-educated Old Believers were also among the 15,424 Latvian inhabitants who were deported to Siberia in cattle trains on June 14, 1941. World War II and the economic, political and atheistic ideology of the Soviet regime impacted heavily on the Old Believer community: private property was nationalized, churches were subject to strict control and spiritual leaders were continually monitored etc. As most Latvian Old Believers were rural residents, collectivization destroyed their usual way of life. Many of the Old Believers moved to cities, and having been isolated from their spiritual leaders, came under the influence of atheistic propaganda. Sociological research undertaken in the 1960s–1970s provide data, that only 2% of the Old Believers at that time were from 18 to 25 years of age (Podmazovs, 2001, p. 150). A decisive role in the secularization of the Old Believers was played by the disappearance of the three-generation family. The older generation were the preservers of the religious traditions in the families of Old Believers, especially the grandmother. When the young people left home (studies, army service, or relocation to cities), they lost their connection with family traditions.

The restoration of the independence of the Republic of Latvia (1990), with the ensuing democratization of society, opened up new opportunities and at the same time created new challenges for the Old Believers community. The Old Believers lacked spiritual leaders for their community, as institutions of religious education had not been operational until 1989. These difficulties were gradually overcome with the establishment of education institutions and the opening of libraries. In Latvia, the Old Believer community stands out with its particular interest in its history^5^: they regularly hold academic conferences and have invited historians from Poland, Great Britain and Russia to read public lectures, as well as supporting academic research in Latvia.^6^

Nowadays, the number of Old Believers in Latvia is significantly smaller than prior to World War II. A reduction in the number of Old Believers can be observed from year to year due to the aging of population, labor emigration, secularization and other factors. For example, from reports submitted by religious organizations to the Ministry of Justice, there were 53,383 Old Believers in Latvia in 2016, but in 2018, their numbers were reduced to 46,482 (Tieslietu ministrija, 2019). Even though the Old Believers form only about 3% of population, this ethnic and religious community has a significant role in Latvian society. In Latvia there is no state religion, nevertheless in 2000 the Republic of Latvia concluded agreements of cooperation with eight Latvian religious organizations (Evangelical Lutheran, Roman Catholic, Orthodox, Old Believer, Methodist, Baptist, Seventh Day Adventist churches and the Jewish religious community). The Old Believers community addresses today’s social problems, including alcoholism, depression, suicide, drug abuse, crime, divorce, poverty and welfare dependency. The representatives of their community are involved in the current public debate, criticizing the encroachment of liberal ideas, particularly—the growing social acceptance of same-sex relationships.

There are more than 70 Old Believer congregations in Latvia (Tieslietu ministrija, 2019). The largest of them is in Riga and has about 5,000 members^7^ (Pazuhina, 2019). It is the only Old Believer congregation in the capital city, while smaller communities exist in the other largest cities in Latvia. Latgale is the most densely populated by Old Believers. There are six Old Believer congregations active in Daugavpils. There are about another 10 Old Believer churches in the near vicinity of Daugavpils. The concentration of Old Believers provides not only an opportunity for them to keep their religious traditions alive and to preserve their cultural heritage, but also creates certain competition between congregations.

## Methods

### Site Selection

Ethnographic data for this paper is drawn from a larger-scale H2020 collaborative research project “Cultural Heritage and Identities of Europe’s Future” (CHIEF) aimed to build an effective dialogue between different stakeholders in order to facilitate a future of Europe based on more inclusive notions of cultural heritage and cultural identity. In this project, cultural identity is understood as a process which emerges through social dialogue, rather than a fixed feature of an individual or group (Hall, 2000). The project is particularly attentive to situational differences in social interactions, as these sharpen understanding of how social agency brings new meanings to young people’s cultural knowledge in defining their identities. For this overarching project, 18 ethnographic case studies were conducted by the consortium partners in nine countries: United Kingdom, Latvia, Croatia, Georgia, Turkey, Spain, Slovakia, India and Germany. In Latvia, this research opted to focus on the Old Believer youth group in Daugavpils, the second largest city of country.

An Old Believer youth group in Daugavpils, a city near Latvia’s southeastern border, with less-developed infrastructure than the capital city and where the unemployment level has reached 8.6% (Nodarbinātības Valsts aģentūra, 2020), was selected for the research. Choosing Daugavpils was based on its multi-ethnic context: 89,662 residents (Pilsonības un migrācijas lietu pārvalde, 2020) of whom 49.0% are Russians, 19.9% Latvians, 13.5% Poles, 7.6% Belarusians, 1.9% Ukrainians, 0.9% Lithuanians, 0.4% Roma and 6.8% other ethnicities, including where no ethnicity was indicated (Centrālais Statistikas birojs, 2018). The large proportion of Russians determines the dominance of the Russian language in the city, and today the Russian language is also the instrument for mutual communication and the passing on of cultural traditions for the Old Believers.^8^ The political orientation of Daugavpils’ Old Believers is determined to a large extent by media. As shown by research on media literacy in Latvia, 90% of the surveyed Latvians usually chose media in Latvian, whereas 80% of the people of other nationalities that were surveyed, usually chose Russian media in Russian for obtaining information (Latvijas Fakti, 2017).

The research was undertaken in Vecā Forštadte (Old Forstadt), a suburb of Daugavpils. This is a suburb of private houses which developed prior to World War II, where a significant number of Old Believers still reside. Apartments which were built in the city suburbs during the Soviet period were not allocated to local residents, as they had been built for immigrants, i.e., the workforce from various USSR territories.

The Vecā Forštadte congregation has about 2,000 members. As in other Old Believer communities, the most active members of the congregation, who regularly attend the church services and observe fasting and other religious traditions, are older than 60 years. The leaders of the congregation are *batushka* and *matushka*, who are among the youngest leaders of the Old Believers in Daugavpils (at the time of the fieldwork—younger than 40 years of age). Whereas the chairperson of the congregation is an active 47-year-old man. The Sunday morning church services in Vecā Forštadte are attended by approximately 40–50 persons, but the numbers increase significantly at special church festivals.

### Data Collection and Analysis

The ethnographic research was carried out over a period of eight months (from June 2019 to January 2020) in the Old Believer youth group in Daugavpils (Latvia). Participant observation, field notes, qualitative semi-structured in-depth face-to-face interviews and technology-afforded tools were the main research instruments used for the data collection. The period of observation varied: from one and a half hours to ten hours. The researcher’s diary was filled in immediately after the observations were made, and the data contained in it were used for analysis.

The Old Believers are a relatively closed and ethnically homogeneous group, which is joined by young people mainly through the encouragement of their parents. In order to arrive at objective research outcomes, the research involved observing the Old Believer youth in their cultural setting. The activities of Old Believer youth are centered on the Sunday school. Observations were made mainly during these Old Believer youth group activities (classes after the church services, excursions to Old Believer historical sites, services at cemeteries etc.). During these group events, the researcher mainly adopted the position of “observer-as-participant” (Johnson and Christensen, 2014). The researcher’s main role was to collect data overtly, and hence the group was aware of the observation activities taking place. Observations were documented in the field diary. While participating in the group events, it was difficult for the researcher to take field study notes, nevertheless the observations were documented shortly after the event.

When designing a qualitative sampling plan, it was estimated that this ethnographic research should require about ten interviews to obtain sufficient data to describe the group and address the research questions. The adding more interviewees would not result in additional perspectives or information because the qualitative research interview seeks to describe the meanings of central themes in the life of the interviewees, i.e., the main task in interviewing is to elicit the interviewees’ experiences, perceptions, thoughts and feelings. Two adults (gatekeepers) and ten young people aged from 13 to 24 years (six males and four females) were interviewed. The gatekeepers were essential for accessing the research setting and group participants. Before commencing the fieldwork, the researcher contacted them and presented objectives and tasks of the project. The gatekeepers were the key consultants to the researcher and informants about the upcoming group activities.

The process of getting to know potential interviewees took place gradually, starting with young people in the family of the religious community’s leader, with the former making up the core of the Old Believer youth group. The researcher was also able to get to know the less active group members by taking part in the Sunday school classes and using the snowball method. The main research instruments in the fieldwork were observation of participants and interviews. The interviews with gate keepers took place as unstructured interviews. This allowed the interviewees to speak broadly and to express their opinion on problems which seemed important to them. The information provided by gatekeepers was primarily related to the history of the Old-Orthodoxy and the community of Vecā Forštadte. The interviews with the gate keepers provided the opportunity to get to know the group’s value system which is passed on to the younger generation. Semi-structured interviews were used with the Old Believer youth. The interviewees provided broad-ranging answers, with extensive discussion about family traditions and celebrations, whereas the thematic range of questions about ethnic and national identity proved difficult. At times, the gathering of data was made difficult by the interviewees’ lack of experience in answering questions when the discussion was being recorded, as well as the low self-esteem of some interviewees.

The interviews took place in Russian and were transcribed following the generally accepted rules for transcription and noting down particularly vivid emotions or longer pauses. The manual by Dresing et al. (2015) was employed when formulating the transcription rules to be followed. The transcribed interviews and the observation field notes were coded using the NVivo software. Coding and analysis were carried out following the grounded theory approach (Charmaz, 2014). To begin with, an interview was selected at random and inductively coded line by line. The findings were discussed with a second researcher and a coding tree was developed as a result of the discussion. The rest of the data was then coded deductively by a single researcher. Emerging ideas, discovered relations and up-coming questions were subsequently documented in the form of memos. If new categories emerged from the data, they were coded inductively and the coded three was adjusted. Each relevant category was then analysed, seeking out common tendencies, controversies and contradictions as well as relations between the categories.

## Results

### Description of the Youth Group

The Sunday school formed the organizational base for the group. It had been set up at Vecā Forštadte and functioned as the Old Believer youth center. It was the main instrument by which the young people were brought together and introduced to the religious and cultural heritage of the Old Believers. The young people got to know each other at the Sunday school and formed informal groups of friends:

Sunday school, as a rule, is set up at those churches (…) where the spiritual father teaches children at an age at which they have to go to Sunday school, that is, let’s say—older than five and under 16. (…) At the same time, you create a certain circle of peers for your children (…) then this group, it will continue to exist as time moves on, and in adult life, at least, sometimes couples get married (Augustin, male, 47 years old, group leader, September 10, 2019).

As to the number of members in the Old Believer youth group: about 35–40 young people took part in various events during the fieldwork. Sunday school was freely accessible to various social groups, but it was closed to members of other religions and Christian denominations. The Sunday school leader expressed his conviction that a child of another religion would feel confused: “A child will not just sit, well, just sit and listen, he will not be interested. (…) now everyone starts praying, and what will he do?” (Augustin, male, 47 years old, group leader, September 10, 2019). The young people expressed a similar view in their interviews—representatives of other religions or denominations should be attending their own Sunday school. A person’s desire to attend the activities with the goal of converting to the Russian Orthodox Old-Rite Church was mentioned as the only way that a representative of another religion or denomination could attend the Sunday school.

The group was homogeneous in its ethnic composition, i.e., young people indicated Russian as their ethnic identity and mentioned the Russian language as the one used within the family. Two of the interviewees indicated that Latvian (in communication with one of the parents or grandparents) was also used in parallel in the family. The other interviewees explained that they were learning Latvian at school. The interviewees have learned the official state language well, due to the gradual transition to teaching in Latvian at state schools (in the interviews, which took place in Russian, one of the interviewees used several terms in Latvian). However, worries were expressed in several of the interviews about the difficulty of learning STEM subjects in Latvian.

The participants of the Sunday school were aged from 5 to 25 years. Preschool and primary school children (from 5 to 12 years of age) did not usually attend church services or just attended a part. They were brought to the Sunday school activities by their parents or grandparents. It was specifically the older generation, especially the grandmothers, who wanted their grandchildren to be “real Old Believers”. Young people from 13 to 18 years of age were the most active group, as its participants (only the boys) help in the organization of the church services and the execution of the religious rituals, alongside the Sunday school activities. The behavior of the young people aged 15–16 years indicated the ones for whom the Old-Orthodoxy was a component of their identity and the ones for whom it was just a formality, and this influenced the regularity of attendance at Sunday school activities. Young people from 19 to 25 years of age independently made the decision to attend the church. If they stayed for the Sunday school activities after the church service, then they joined in with the activities taking place in parallel for the adults. The regular attendance at group events by this age group is made more difficult, as many of them are studying and also work while studying (mainly in the services sector, where employment also includes Sunday work).

A particular single gender numerical predominance cannot be observed in the Old Believer youth group,^9^ but there is a strict division of gender roles, as boys and girls have different obligations in the Old Believer community. There is a close correlation between religion, ethnicity and gender in the Old Believer community. The young people did not perceive any threats from mixed families, but the leaders of the Old Believer community expressed concerns that having family members belong to different denominations led to conflicts and a loss of religious identity:

Marriages of people belonging to different denominations existed, exist and will exist, nothing can be changed in this regard (…) then it’s very difficult to determine how to baptize [a child], because no one wants to meet each other half way in this case, and this is a conflict. (…) even if one of the parents wins, it’s still a conflict later on, it smolders inside, and here only the weak religiosity of one of the parties can cure the situation (Augustin, male, 47 years old, group leader, September 10, 2019).

An explanation by a second leader confirmed that the passing on of religious traditions is determined by gender, respectively, the female was considered to be the main preserver of religious tradition and the one to pass it on to the younger generation:

(…) and the worst thing is when a child is baptized in a different faith, not in his mother’s one. For example, it often happens that the mother is an Old Believer, but the child is baptized in a different faith, in his father’s. As the child is growing up, he doesn’t know either the faith he was baptized in, nor his mother’s faith, and he’s at a crossroads, he doesn’t know where to go, he’s a stranger there and a stranger here, because he knows nothing (…) Adults must understand who will deal with the spiritual matters of the child’s life. This is the main issue (Sergey, male, 39 years old, group leader, September 12, 2019).

There is a strong hierarchy at the core of the group structure: its operations are coordinated by the congregation’s chairman and the spiritual leader, while the functions of intermediary between the leader and group members is undertaken by the spiritual leader’s wife, who is the one who receives requests, demands and proposals. The young people noted that there is not, and cannot be, an informal leader. Observing strict subordination, even the most active group members never expressed their initiative in organizing an event, but always undertook the tasks entrusted to them by the adults. The four most active boys in the group (Fyodor, Boris, Dima and Yoan) formed the core of the Old Believer youth group. A certain type of hierarchy also held sway between them: the oldest one (Fyodor) received tasks from the chairperson of the congregation or spiritual leader and then tried to involve the other young people in the completion of the tasks. As explained by the interviewee, this was not simple:

(…) the assignment caused great difficulties, because it was complicated to find a person at school able to fulfil this task. First, although there were a lot of Old Believers in our school, they were not ready to help me. Second, everyone was very busy and did not want to spend time on some church, as they put it (Fyodor, male, 16 years old, school student, September 19, 2019).

The most active young people in the group are also the most regular attenders of Old Believer group events. The number of people attending Sunday school can be mentioned as the main indicator for involvement in group activities: this number dwindled over four months, from 40 in September to 2–3 in December. Furthermore, there was a higher indicator of attendance in activities away from the church (excursions and services at the cemetery) or at events where there were presents expected for those attending (on 1st September and at Christmas).

### Competing Identities of Old Believer Youth

In the context of ethnic diversity, in Latvia, as in other countries “the development of identity takes place against the backdrop of the broader society and the value that society places on one’s social group membership” (Cheon et al., 2020, p. 1). In the case of Old Believer youth, an awareness is developing that they are members of several social groups. Their ethnic community, religious congregation and Latvian society influence the self-identity of Old Believer youth significantly, i.e., in how they identify and define themselves. Ethnic, national and religious identities, as dimensions of social identity, form a unique configuration, by which Old Believer youth differ from peers of their own age. The majority of research on identity has been focused on ethnic identity, national identity, followed by racial identity. The religious identity has been largely neglected in the qualitative research approach literature. Moreover, religious identity is not operationalized in the studies. The concepts of religiosity and spirituality are often used but they differ from religious identity which emphasizes a social identity involving membership in a specific religious group. We will be focusing attention on the subjective perceptions of the young people’s identity multiplicity, based on the qualitative interview data.

#### Youth Understanding of Concepts

It should firstly be noted that marked difficulties appeared in the responses of the interviewees in their understanding of concepts like “ethnic identity” and “national identity”, which were mainly caused by two factors. The first is that ethnic and national belonging are not important issues for the young people, which is confirmed, for example, by the following response: “I don’t think about what ethnic group I belong to (…) I treat all people equally, regardless of who they are—Latvians, Russians, it doesn’t matter to me at all” (Avakum, male, 18 years old, school student, December 12, 2019). Secondly, an understanding of the mentioned concepts is influenced by the age peculiarities: issues of identity turned out to be very difficult in the age from 13 to 18 years, and even in the age from 19 to 25 years, some interviewees posed the question to the researcher of what the difference was between ethnic and national identities. During the interviews, it appeared that when the term “nationality” was used, the young people often thought it was “ethnicity,” for example, Alex expressed himself as follows:

Probably, it [ethnicity] manifests itself through the fact that most of my friends and acquaintances are Russian-speaking, and I communicate with them in Russian only. It also manifests itself through the word “Russian” printed in my passport in the section for nationality (Alex, male, 22 years old, employed, October 16, 2019).

It should be clarified that an entry for ethnicity had not been included in passports in the Republic of Latvia, but this changed after the occupation of Latvia in 1940, with the practice being introduced on the issuing of Latvian SSR passports. The inclusion in a passport of an entry for ethnicity, does not conform to the Republic of Latvia’s principle of continuity or international practice. However, since 2013, the entry “tautība” (ethnicity) can be included in a passport in the Republic of Latvia, indicating a person’s ethnic identity, if a person so requests. Bearing this context in mind and returning to what Alex stated, we can conclude that the interviewee was clearly conscious of his belonging to an ethnic community, and also wished to demonstrate this by an entry in his passport.

#### Maintaining Religious Identity

The Old Believer youth describe self-understanding as a composition of intersecting identities that influence each other. An interviewee, in discussing her identity, first mentioned her ethnic identity, which was then followed by an allusion to religious identity, which was passed down in the family:

First of all, my family is Russian, we’re Russians. And yes, perhaps an important aspect is that we’re Old Believers, because in my family, along my family line in particular, there are no other religious faiths in my immediate family. There are no Catholics, no Orthodox, all the family line consists of Old Believers (Feodosiya, female, 24 years old, student, December 27, 2019).

Religious identity is the central axis around which other dimensions of social identity are formed for Old Believer youth, as Old Believers have found stability in religion for generations, regardless of differing social systems (the tsarist empire, the Soviet regime and democracy), and religious belonging is allocated an important role in today’s globalization as well: “Yes, I’m an Old Believer; and the Old Believers in Latvia, and not only in Latvia, are trying to preserve their identity despite political and cultural changes at the place where they are” (Alex, male, 22 years old, employed, October 16, 2019).

More than one interviewee emphasized that religious identity is more important than ethnic or national identity, but at the same time it was explained that one’s religious belonging did not have be demonstrated to society:

I don’t think that nationality is the most important thing for people, religion is very important for a person. For me, of course, it’s important that I’m an Old Believer, that I go to the prayer house. It’s my personal business, I don’t display it to others (Yoan, male, 14 years old, school student, November 3, 2019).

The position that religious belonging is a private matter and does not have to be demonstrated openly within society, has helped the Old Believers to survive stigmatization for several centuries. The Orthodox turned sharply against them for their religious conviction, the communists tried to reeducate them, and in today’s secular society too, the Old Believers are still, to a certain degree “the odd ones out.”

The interviewees create their Old Believer identity, based on their belonging to the religious group, which is strengthened by the fact that they value this in-group more favorably than the out-group. One interviewee has the conviction that the Old Believer denomination is the only true Christian Church:

They [parents], my dad, to be more precise, helped me understand what faith is. He gave me several, many pieces of convincing advice. But I think, most likely, everything depended on me, because when I saw what Sunday school was like, and faith in general, I immediately understood that I had to participate in this. I had to attend Sunday school, perhaps because my conscience also longed for this place. I tried to think in a bigger picture because many people are non-believers, yes, and faith—it’s a complicated notion. And this faith, that is, the Old-Orthodoxy, the Old Believers, seemed to me true (Fyodor, male, 16 years old, school student, September 19, 2019).

The religious community provides a sense of belonging and acts as a central source of pride and self-esteem. Belonging to the Old Believer community is the central element in the identity of the interviewee, which is why many cultural expressions are evaluated through an Old Believer prism. Something is only considered to be correct, if it corresponds with Old Believer norms. The interviewee stated that his religious belonging was a hurdle to the common celebration of festivals at school, which have recently entered the Latvian cultural environment:

But at school… Festivals… I think it’s a problem for some denominations, because Halloween or St. Valentine’s Day are festivals an Old Believer mustn’t celebrate together with others. Since they’re invented and, generally speaking, they’re not associated with anything sacred (Fyodor, male, 16 years old, school student, September 19, 2019).

#### Issue of Ethnicity

It should be emphasized that two dimensions of social identity, the ethnic and the religious, are the most closely and tightly knit for Old Believer youth. The interviewees see themselves as Russian Old Believers and are trying to preserve their ethnic-religious identity despite various political and cultural changes. The Russian language is the instrument that integrates these two dimensions of social identity tightly. One has to take the language policy, which has been implemented in Latvia into account, to gain an understanding of the attitude of the interviewees towards their native language.

Since 2004, a bilingual education model has been implemented in minority schools to ensure that all children and young people have an equal access to high-quality educational opportunities in Latvia. Since 2019, there has been a gradual transition towards teaching in Latvian, with the goal of moving to teaching in Latvian completely at the general secondary education level, by the 2021/2022 school year. Furthermore, three models are being offered at the general basic education level, which provide for teaching in Latvian for 50–80% of school subjects, starting from the 2021/2022 school year. The interviewee does not support this language policy and interprets the gradual transition to teaching in Latvian as the closing of “Russian schools”:

There are no problems in the city [Daugavpils] itself, I understand that there are two large cultures that can exist in Latvia: Russian culture and the culture of the country, that is, Latvian culture. In the city itself there are no contradictions in this regard, but the state, the state closes many Russian schools, I think this is like a conflict (Avakum, male, 18 years old, school student, December 12, 2019).

Such an interpretation provides evidence about a sharpened perception of language policy, which is rooted in an understanding of ethnicity. Respectively, language is considered to be the main indicator of ethnic belonging. Therefore, the transition to teaching in Latvian is considered as a threat to the existence of the ethnic minority.

The Russian youth do not see any point in learning Latvian, as it is difficult for this language to compete with other languages in the free market situation:

Let’s say, the same English is now very necessary for employment even in Latvia, and in the European Union, English is very important. Yes, foreign languages are in general important. Let’s say, I arrive in a country and I can communicate in English almost everywhere, I think someone will understand me there anyway, yes. But, of course, Latvian can be used only in Latvia, it’s unlikely that Latvian will be understood in any other country, I also think that Russian will also be understood in many countries. German, probably, is also very important (Yoan, male, 14 years old, school student, November 3, 2019).

The importance of a foreign language, especially English, was highlighted by other interviewees, who also pointed out the increasing role of English in Europe and elsewhere in the world. Furthermore, they view this positively, as knowledge of English provides an opportunity for studying at universities and getting an education in other countries:

For me, it’s positive that thanks to English you can study in other countries. There are universities in other countries whose official language is not English, but in which you can study in English and acquire the education of that particular country. That is, English, it really provides very great opportunities, opens many doors to you. Even when you just, when you need to talk to someone in another country where they don’t know your native language, most likely they know English, which is considered an international language (Avakum, male, 18 years old, school student, December 12, 2019).

Even though this interviewee stated that his own ethnic identity, and that of his friends, acquaintances and people around him, was of little importance to him, he was deeply offended by the lessening of the role of Russian in Latvia, although he viewed the increasing role of English around the world positively, and saw no threats in this.

An interviewee who regularly takes part in cultural events where the participants belong to different ethnicities and various religions, and also communicate between themselves in various languages, emphasized that language is a very important instrument of communication:

They [languages] are very useful, because if we live in Latvia, we must know the Latvian language. You can communicate more, of course, in Russian, but in Latvian you will find more information immediately, learn more immediately, because everyone starts answering right away and it’s easier for you to switch to Latvian than to try answering in English, but also English—it’s a universal language you need everywhere to understand other people (Olga, female, 22 years old, employed, December 10, 2019).

#### Complexities of Nationality

National identity is an important dimension of social identity in addition to religious and ethnic identity for Old Believer youth in Latvia. Young people have experienced the importance of having Latvian nationality in contact with people from other countries, for example, when visiting there:

A: It [nationality] manifests itself when I, for example, travel round the world: I participated in the ERASMUS programme, and I emphasized that I was from Latvia. Everybody was interested to hear about Latvia, about the Latvian language. And recently a friend from Russia visited me, she was also interested in learning more about Latvia, because I’m a sort of her friend from Latvia. It’s interesting to learn about the Latvian nation, about the Latvian language from a person who lives in this particular country. That is, on the contrary, I think it helps more than… Q: That is, do you feel you belong to the Latvian nation according to your inner conviction or because you are considered to belong to it? A: According to my inner conviction (Olga, female, 22 years old, employed, December 10, 2019).

On the other hand, members of ethnic minorities within Latvia often associate national identity with *Latvianness* (or being like a Latvian) as an ethnic category. Therefore, in defining their national belonging, they avoid using the term *латыш* (in Russian) or *latvietis* (in Latvian), meaning “Latvian”. They prefer to use the neologism *латвиец* (in Russian), *latvijietis* (in Latvian), meaning “one living in Latvia.” This is an artificially constructed term, which has been used regularly in recent years by media that write or broadcast in Russian, so that, when nationality is being discussed, ethnic Latvians and representatives of ethnic minorities are not all referred to by the same term—“Latvian.” Taking this socio-political context into account, this type of self-identification by the interviewees is not surprising:

1 I am a *латвиец* [person living in Latvia], I am a citizen, I live in this country (Alex, male, 22 years old, employed, October 16, 2019).
2 There are *латыши* [Latvians] and there are *латвийцы* [people living in Latvia] (Avakum, male, 18 years old, school student, December 12, 2019).
3 Q. Do you consider that Latvia is a part of Europe? A. Yes. Q. What is an inhabitant of Europe? A. *Латвиец* [person living in Latvia] (Dima, male, 13 years old, school student, October 13, 2019).

This position from the interviewees indicates that ethnic identity is more important than national identity to them, with a neologism that differentiates ethnic minorities from Latvians, being used to describe it.

In the statements from the other interviewees, it was not the attitudes and beliefs about their ethnic group belonging that were revealed, but rather their efforts in thinking and searching for meaning in their belonging: “I think, I’m Latvian, because I’ve been living in Latvia for my entire life, I’m just a Russian speaking Latvian” (Marfa, female, 20 years old, employed, December 16, 2019). This is a significant example of a mixed family: the interviewee communicates with her father and other members of the family in Russian, but with her mother—in Latvian. This has influenced her self-identity: the interviewee recognized her belonging to Latvia and was not afraid to call herself a Latvian. She avoided the use of the neologism *латвиец* (person living in Latvia), even though she mentioned the geographical location of her living space as the main criteria of national identity, and not her socio-political belonging, i.e., citizenship.

Another interviewee also expressed a similar position: “Since I live in Latvia, I should probably relate myself more to Latvians, I’ve been here since my birth” (Yoan, male, 14 years old, school student, November 3, 2019). The word *probably*, which the interviewee included in the response, as well as the intonation of his voice, provided evidence that he was not completely convinced about his answer. The basis of this uncertainty is the artificial segregation by Latvian society into Latvians and Russian-speakers, which usually becomes more acute prior to the parliamentary elections.

However, the responses of these interviewees were marked by the fact that they understood the concept of “nation” to be a political category and associate their belonging to the country through this. It should be emphasized that the problem of citizenship for Russian-speakers did not surface in the interviews with the Old Believer youth, as due to having been citizens of the Republic of Latvia prior to 1940, the Old Believers and their descendants regained their citizenship status after the renewal of Latvia’s independence.

#### Sense of Being European

The question of European identity has been particularly significant since Latvia joined the European Union (2004). In relation to the Old Believers in Latvia, we could ask how young people from ethnic or religious minorities experience their belonging to Europe? The interviewees found it hard to define what a European is, and what characterizes European culture. They doubted whether the older generation (born during the Soviet period) and immigrants from other continents, for example, could be considered as Europeans. The geographical area, and being rooted in European culture, were usually mentioned as the determining criteria for European identity. Furthermore, culture was specifically indicated to be the determining factor: if someone had no knowledge about European culture and did not practice European traditions, they could not be considered to be Europeans:

A European, perhaps, first of all, is a person who lives in Europe, so to speak, by location, by place of residence. And the one whose roots go deep, who is connected with European culture in particular. (…) A person who arrives in Europe from somewhere else and settles here, I, in principle, don’t consider him European, because he does not really know and practice European traditions (Feodosiya, female, 24 years old, student, December 27, 2019).

Other interviewees also highlighted this cultural belonging. It was emphasized that to be European, means accepting European values:

(…) “to be” a European means to know more, a little bit more than others, about Europe in general, about various interesting topics as a whole. And “not to be” means, for example, not being tolerant towards something, not being educated in some kind of field and making a judgement about a person by, for example, by… Making a judgement about a person by his looks (Olga, female, 22 years old, employed, December 10, 2019).

When asked to explain what European values were, interviewees indicated that the European value system was based on fundamental achievements of democracy*,* like religious freedom, cultural diversity, multilingualism, human rights and tolerance. At the same time, the young people spoke about negative features pertaining to Europeans: Eurocentric thinking (defending European interests without respecting the specific character of other cultures) and materialism: “That is, we’re sort of drawn into a materialistic way of life compared to Japanese or Chinese who are more spiritual” (Avakum, male, 18 years old, school student, December 12, 2019).

Some interviewees admitted that they are Europeans when discussing their personal belonging to Europe, because they live in Latvia which is a European country, and they have a European mentality. In contrast, others admitted that they do not completely feel like Europeans for several reasons. They are not part of the European information space, they feel a greater belonging to the culture of the region (Latgale) than to Latvian and European culture, they feel a greater belonging to their ethnic, respectively, Russian culture. Furthermore, the young people’s understanding of what a European is, was characterized by a connection with Latvian language skills:

Q: Who is a European in your opinion? A: I think a European is a person who lives in Latvia, knows Latvian. (…) Q: Do you consider yourself a European? A: No. Q: Why? A: Because my Latvian isn’t very good. Q: What does it mean for you not to be a European? A: I don’t know (Tanya, female, 14 years old, school student, November 10, 2019).

European identity was associated firstly with the European Union for the interviewees, in the political system where they see both advantages (the opportunity to travel freely and gain an education in the European Union), as well as shortcomings. That the young people perceive that there are more shortcomings than advantages has to be admitted. They consider that Latvian politicians are unable to protect their country’s political and economic interests: “I think that Latvia has to be in the European Union, but it needs to have its own view on matters, it just seems to me that, in the European Union, it listens more to the opinions of other countries and forgets about the interests of its people” (Olga, female, 22 years old, employed, December 10, 2019). The mentioned shortcomings of the European Union included travel to Russia and Belarus (visas are required and there is strict border control, which the interviewees considered to be a hurdle for their own way of life, as well as for cultural and economic development of Latvia).

It is significant that the European Union is perceived as a threat to the maintenance of unique cultures in the long term: “In my opinion, the culture of every nation gets erased if the country is part of some kind of commonwealth” (Alex, male, 22 years old, employed, October 16, 2019). The young people were dissatisfied with the dominance of European goods over local products and products from the border countries (Russia and Belarus): “If people live in Europe, then of course it’s clear that goods come mainly from the European Union. From other parts, there are somehow less goods and, well, in shops, respectively” (Yoan, male, 14 years old, school student, November 3, 2019).

Even though the interviewees expressed criticism of the European Union, they still maintained that being the European Union member state was the best choice for Latvia. Therefore, it was surprising that several interviewees did not associate their future with the European Union. In their responses, there was a marked desire to move to live in other countries:

(…) I really love this country as a location. But I can’t say that I love this state to the same extent, no. (…) life is bad and hard, and you can do something only in your personal life and family, that is, to go somewhere (Alex, male, 22 years old, employed, October 16, 2019).

Due to their ethnic belonging, Russia was most frequently mentioned as a place to live in the future:

Perhaps I would go to Belarus or to Russia, since I have visited these countries, I know Russian well, (…) and these countries are closer in terms of culture, although I, if I could choose, I’d prefer to leave for Russia, because (…) there are the Old Believers in Russia and associations that suit me fine (Fyodor, male, 16 years old, school student, September 19, 2019).

The current study’s findings demonstrate that several dimensions of social identity (ethnicity, religion and nationality) are inextricably intertwined for Old Believer youth. Furthermore, the configurations are quite disparate. Some interviewees persuasively highlighted their ethnicity and precluded blending into mainstream *Latvianness*. The others maintained that their feelings of *Latvianness* existed in parallel with their belonging to an ethnic minority. Others, though denying their *Latvianness*, still expressed a desire to enrich their ethnic identity, selectively choosing to acquire the culture of another ethnic community (mainly English). As identity is a social construct, a decisive role in its development is the young people’s perception of the opportunities in their future, which are influenced by socio-economic and educational factors.

## Discussion

The Old Believer community in Latvia has been strongly influenced by the rapid socio-political transformations of the twentieth century: two world wars, forced collectivization and industrialization, nationalization of property, the regaining of independence, denationalization of property, a notable population decline, the emigration of the workforce etc. As a result of all these changes, the Old Believers have moved from being quite an isolated community, residing mainly in rural areas, to a community residing in a semi-urban or urban environment. The clash between the traditional and the modern way of life is still reflected in the search for identity of the younger generation of Old Believers.

Certainly, today’s rapid changes are leading to identity crisis: an individual faces difficulty in shaping and maintaining a stable identity, since economic life is becoming increasingly unpredictable and communities are becoming fragmented. The fact that young people’s searches for identity are becoming increasingly more complex has been expressed by several researchers on young people (Erikson, 1980; Marcia and Archer, 1993; Côté, 2009), emphasizing that extended searches for identity can facilitate their marginalization (Côté, 2009). The identity of Latvian Old Believer youth forms and develops across multiple contexts, and is strongly affected by the social transformation: changes in cultural symbols, rules of behavior, social organizations and value systems.

The qualitative data collected during the fieldwork provided a useful resource for an analysis of belonging, the crucial factor in the formation of identity for Old Believer youth. As the voices of young people in this study reveal, three types of belonging characterize Old Believer youth: their ethnicity, which interacts with national belonging in a complex way; the local belonging, which is stronger than the global one; the European belonging, which conflicts with national belonging and ethnicity.

As stated before, religious identity is the central axis around which other dimensions of social identity are formed for Old Believer youth. In the process of identity formation, Bisin distinguishes two types of mechanisms: cultural conformity and cultural distinctiveness (Bisin et al., 2016). Cultural conformity means that minority groups adopt inclusive identities and that they integrate into their social surroundings. Whereas cultural distinctiveness means that minorities keep their identities and reduce interactions with individuals from other ethnic groups. The Old Believers are markedly characterized by cultural distinctiveness, as they had lived in quite isolated communities so that they could sequester themselves from other religious groups, right up until the end of World War II. This isolation from others has been partly maintained even today. It has been established that values determined by their religion are more important for the identity of Old Believers than ethnic characteristics.

The religious identity, as performative identity (Butler, 1997), manifests in two different spheres: within the community as a group, and outside the community, as a minority which differs from mainstream society. As shown by data from the interviews, the young people do not wish to demonstrate their religious belonging outside of their community. There are several reasons for this: firstly, displaying their religious belonging in a public place, for example, at school, would be a challenge to secular society. On the other hand, by not highlighting their belonging to the Old Believers, the young people are trying to avoid social marginalization, as the Old Believers have felt prejudice for centuries against them from the Orthodox people, something that has not disappeared as yet. Thirdly, religious belonging is associated with religious feeling, which is an internal individual experience. The religious identity is a carefully guarded value for the Old Believer youth, which must be protected from the eyes of strangers.

In present day Latvia, Old Believer religious identity is an “added” value for Russian ethnic belonging. It should be clarified that the Soviet regime caused a rapid demographic shift in Latvia. Before World War II, there were 75.5% Latvians in Latvia (Salnītis, 1936, p. 288), but in 1989, Latvians only constituted 52% of Latvia’s population (Latvijas, 1990, p. 6). These demographic changes were caused by forced industrialization. The arrival of a workforce from Russia and other Soviet republics, together with a policy of Russification caused historical trauma among Latvians, which has yet to heal. Respectively, bias against Russians is quite characteristic for Latvians with right-leaning views. At the same time, a trend can be observed for Russian immigrants from the Soviet period to be differentiated from the Old Believers who arrived in Latvia much earlier, as they are considered “our Russians”, i.e., they speak Latvian, are trusted and loyal to the Republic of Latvia. In this way, ethnic identity gains a different social evaluation due to religious identity, allowing for the avoidance of individual indirect discrimination.

Even though the interviewees’ identities are variable, and not clearly ascertained or defined, there are clear lines beginning to emerge: the Old Believer youth are characterized by a powerful consciousness of ethnic belonging (Russian), and equally also, their belonging to a religious community (Old Believers). The actual complexity of multiple identities is reflected in the young people’s subjective representation of their identities. From one side, the young people who are both Old Believers and Russians maintain a relatively simplified identity structure: they think of their religious group as composed primarily of Russians, even though, objectively, there are non-Russian Old Believers in Latvia as well. In other words, they perceive the different groups to which they belong as containing the same members.

Furthermore, both dimensions, ethnic and religious, are also covered by a territorial aspect—a marked attachment to their place of residence. The interview data show that the young people mainly attend cultural events in their local city, i.e., in Daugavpils, and they are characterized by a feeling of regional belonging to the eastern part of Latvia, to Latgale. Another dimension, although weak, also appears—the global dimension, which is marked by the desire of respondents to study or work in other countries (Germany, the Scandinavian countries and Spain were mentioned) or to move to another country, to live (Russia and Belarus were mentioned).

In the case of the Old Believer youth, national belonging, in other words, a belonging to a national community, is aligned closely with ethnic identity. In other words, the way in which the young people look at their belonging to a nation is related to the way in which they think about their belonging to a certain ethno-cultural community—Latvian, Russian, Polish etc. National belonging, as a sense of political community (Smith, 1991) is quite foreign to the interviewees. The sense of ethnic belonging is marked by the group’s common origins, language, traditions, territory and religion. Nowadays, illusions that ethnic identity will weaken and blend together, along with modernization and democratization, have disappeared. The opposite process is actually taking place and modernization of society is facilitating the strengthening of ethnic identity. It continues to exist alongside the other identities and comes into conflict with them. It would, therefore, be a mistake to evaluate the role of ethnic identity too low, or to look at it as an insignificant or fading phenomenon.

The fact that the young people who were interviewed feel a stronger belonging to their nation than their belonging to Europe is significant. Even though Latvia is geographically located in Europe and is the European Union member state, the interviewees’ have problems in developing a European identity. In Europe, the diversity of national and ethnic culture is considered to be an important foundation for a democratic society. However, researchers admit that it is also the main hurdle to the development of a European identity, as the idea of a supranational Europe is becoming a threat to different cultures and national identities (Angeles Marin, 2003).

In the current European identity research, two approaches are relevant: the instrumental and the cultural (Jiménez et al., 2004; Verhaegen et al., 2014). Nevertheless, the concepts of instrumental and cultural considerations for European identity construction have not been applied to ethnic minorities. The scholars argue that research on different ethnic, cultural, and religious groups still needs to be done (Wallace and Strømsnes, 2008). This analysis of the qualitative data shows that the young people of ethnic and religious minority in Latvia use instrumental considerations for European identity construction. From one side, the researched Old Believers young people see advantages in the EU membership: the opportunities for traveling, benefits of open boarders, study abroad or finding a job, and participation in various youth programs. From another side, they strongly associate negative consequences with the EU membership: social, economic, and ecologic problems. This finding is consistent with the previous research in Lithuania which has shown that perceived threats to their country due to EU membership do not allow the minority of Russians to identify with Europe or the EU (Waechter, 2017). In turn, cultural considerations are particularly important for construction of ethnic identity. The young Old Believers in Latvia have developed their ethnic identity based on cultural considerations, mainly language and cultural traditions. They explained their feelings of belonging to Russia by identifying common culture that distinguishes Latvians and Europeans from Russians.

The fieldwork data reveal that, as understood by Old Believer youth, a “European” is a political category, and the concept of European gets connected with a belonging to the European Union. Respondents (mainly aged 13–18 years) who have not visited other countries, have greater difficulty in describing European culture, their belonging to the European cultural space and an understanding about European values. In contrast, young people (mainly 19–25 years of age), who already have experience in developing cultural contacts, confidently consider themselves to be European and confirm their belonging to the European cultural space. As respondents highlighted that the European Union, as a political structure, threatens Latvia as a small nation in terms of preserving Latvian national culture and defending national interests, we can conclude that European identity is perceived to be conflicting with national, ethnic or regional identity, rather than being inclusive, as envisioned by the European Union agreements and guidelines.

Therefore, European identity is connected mainly with a belonging to the European Union in the awareness of Old Believer youth. The research results can be explained using instrumental theory, which emphasizes that people base their decisions on whether they feel that they belong to Europe or not, on rational arguments: if belonging to the European Union provides them with more benefits than losses, then their European identity develops (Huyst, 2008). At this point, we should take into account here that the level of trust of Latvian population in the European Union is relatively low. In 2002, 46.9% of those surveyed described their attitude as being negative towards the European Union (Latvijas Vēstnesis, 2002), and research data from 2011 revealed that the majority of those surveyed (67%) admitted that they do not feel like the European Union citizens in their daily lives. A *Eurobarometer* survey on the attitude of inhabitants of European countries towards the European Union from 2019, revealed similar results—among the other countries, Latvia has the ninth lowest level of trust in the European Union—25% (Eurobarometer, 2019). The conclusion can be made that the dissatisfaction of Old Believer youth with economic and political aspects of the European Union, which are not facilitating rapid growth in the well-being of the inhabitants of Latvia, create hurdles to the development of their European identity, as the respondents do not clearly sense themselves to be European.

## Data Availability

The raw data supporting the conclusions of this article will be made available by the authors, without undue reservation.

## Footnotes

1 The Old Believers were skillful carpenters. Latvia’s heritage of wooden architecture which has been preserved till today was to a large extent created by Old Believers.

2 Here, and throughout this article, translations are by the author.

3 Nowadays this place is located within the city boundaries of Daugavpils. The Old Believers community of Daugavpils is proud of this fact, and a cross of remembrance was erected there in 2003.

4 The Old Believers were forbidden from filling in registration books of births, deaths and marriage and their marriages were not considered to be lawful. In 1847, a ban was implemented on accepting Old Believers into trading guilds. The Old Believers were denied permission to build or restore any church if an Orthodox church was anywhere in the vicinity.

5 The Latvian Old Believers community has a long tradition of research carried out in Latvia. The works by A. Podmazovs *Vecticība Latvijā* (The Old-Orthodoxy in Latvia), N. Pazuhina and I. Ivanov *Рижские староверы* (Rīga’s Old Believers), Z. Zimov *Старообрядцы Екабпилса* (*Jēkabpils Old Believers*), V. Baranovsky and G. Potašenko *Староверие Балтии и Польши* (*The Baltic’s and Poland’s Old-Orthodoxy*), and V. Nikonov *Староверие Латгалии* (*The Old-Orthodoxy in Latgale*) should be noted as being the most important published in recent years.

6 The Old Believers community finances the publication of the *Latgale Old Believer Dialect Dictionary* *Диалектный словарь староверов Латгалии* prepared by Daugavpils University Professor Jeļena Koroļova, which provides unique material on *Slavic linguistics*.

7 Whereas, in an interview it was mentioned that there were 25,000 members in the Riga congregation (Augustin, male, 47 years old, group leader, September 10, 2019). The significant difference in the numerical indicator can be explained by the fact that the Latvian Old Believers count only those members of the congregation who have voting rights at the congregation’s annual meetings.

8 Young people in the religious congregation are offered the opportunity to learn the ancient Slavic language, Old Church Slavonic (*церковнославянский язык*), which is an important component of the identity of Old Believers.

9 The majority of the middle-aged and older generation congregation members who regularly attended the church were female.
